# Clinical evidence of acupuncture for luteinized unruptured follicle syndrome: a systematic review and meta-analysis of randomized controlled trials

**DOI:** 10.3389/fendo.2025.1640820

**Published:** 2025-08-29

**Authors:** Ruifang Zhang, Guangyao Lin, Wei Wang

**Affiliations:** ^1^ Department of Gynecology and Obstetrics, Affiliated Hospital of Gansu University of Chinese Medicine, Gansu, China; ^2^ Center for Reproductive Medicine, Department of Obstetrics and Gynaecology, Peking University Third Hospital, Beijing, China

**Keywords:** acupuncture, infertility, luteinized unruptured follicle syndrome, ovulatory disorders, meta-analysis

## Abstract

**Objective:**

This study aimed to systematically assess the efficacy of acupuncture in women with luteinized unruptured follicle syndrome (LUFS) based on existing randomized controlled trials (RCTs).

**Methods:**

A search of eight databases and one clinical trial database was conducted on May 3, 2025, to identify relevant RCTs examining the benefits of acupuncture for LUFS. The clinical outcomes of interest included two primary outcomes and five secondary outcomes. Forest plots were used to illustrate the pooled results, and sensitivity analyses were performed to verify the robustness of the evidence. Subgroup analysis was conducted to investigate whether the effect of acupuncture on the primary outcomes was related to the number of acupoints used per treatment. In addition, Begg’s and Egger’s tests were conducted to quantitatively examine publication bias among the studies.

**Results:**

A total of 15 RCTs from China involving 1,030 participants with LUFS were included. According to the pooled results, acupuncture intervention effectively increased the ovulation rate by 25% [risk difference (RD) = 0.25, 95%CI = 0.21–0.30, *p* < 0.00001] and the pregnancy rate by 22% (RD = 0.22, 95%CI = 0.16–0.28, *p* < 0.00001) compared with the control group. Moreover, acupuncture treatment was more conducive to improving the luteinizing hormone levels [mean difference (MD) = 3.76, 95%CI = 2.27–5.25, *p* < 0.00001], the estradiol levels [standardized MD (SMD) = 0.47, 95%CI = 0.31–0.63, *p* < 0.00001], the progesterone levels (MD = 1.50, 95%CI = 1.09–1.91, *p* < 0.00001), the resistance index (MD = −0.07, 95%CI = −0.09 to −0.05, *p* < 0.00001), and the pulsatility index (MD = −0.10, 95%CI = −0.15 to −0.06, *p* < 0.00001) of the ovarian artery. Subgroup analysis indicated a higher ovulation rate with stimulation of more than six acupoints (28%) compared with six or fewer acupoints (19%); however, there was no notable association between the number of acupoints and the pregnancy rate (22% *vs*. 23%). Furthermore, sensitivity analyses confirmed the robustness of the results, while both Begg’s and Egger’s tests indicated no significant publication bias across studies.

**Conclusions:**

This pooled evidence from Chinese RCTs reveals that acupuncture is a promising complementary therapy for LUFS. However, these findings might not be generalizable outside China, and most trials exhibited deficient methodological reporting. Therefore, further research studies with more rigorous designs and larger sample sizes are needed to confirm the efficacy of acupuncture for LUFS.

**Systematic review registration:**

www.crd.york.ac.uk, identifier CRD420251062225.

## 1 Introduction

Infertility is a severe public health concern and affects 9% of women worldwide (1). Between 1990 and 2021, the prevalence rates of infertility have increased by an average of 0.68% among women (2). In 2021, there were approximately 110 million reproductive age women suffering from infertility in many regions (2). Notably, the infertility prevalence was considerably high in most regions, with 12.7% in the US, 25% in China, 24.5% in Kenya, and 14.7% in Uganda (3–5). Emerging data from the French National individual medico-administrative database suggest that the economic burden of infertility accumulated to €70.0 million for 10,000 women (6). On the other hand, among 3,332 infertility-related initiatives, US $52.6 million was targeted for fundraising, of which US $22.5 million was actually raised in the US between 2010 and 2020 (7). The World Health Organization (WHO) has declared infertility a cause of disability, which means that healthcare services for infertility fall within the scope of the Convention on the Rights of Persons with Disabilities (8). Consequently, a substantial number of countries worldwide are establishing health policy legislations to address infertility care (9). For example, Australia, Singapore, Iran, the US, and the UK have established diverse public health financial protection focused on the treatment of infertility (10).

The causes of infertility are greatly intricate, including ovulatory disorders, endometriosis, uterine factors, and tubal occlusion, among others (3). Of these categories, approximately 25% of infertility has been diagnosed as ovulatory dysfunction, with most anovulatory women suffering from luteinized unruptured follicle syndrome (LUFS), which is characterized by mechanical impairment of follicular rupture (preventing oocyte release) despite the occurrence of luteinization and other endocrine features typical of the luteal phase of the menstrual cycle (3, 11). The incidence of LUFS in women with unexplained infertility is approximately 25% (12). In clinical practice, the most common treatment regimens for LUFS include ovulation induction, assisted reproductive technology (ART), surgical approaches, and administration of granulocyte colony-stimulating factor (G-CSF) to improve the ovulation and pregnancy rates (13, 14). Despite their overall effectiveness, a number of ovulation induction drugs such as clomiphene citrate (CC) and letrozole are often associated with reduced numbers of retrieved oocytes and a higher incidence of cycle cancellations in women undergoing *in vitro* fertilization (IVF) (15). G-CSF, as a novel therapy to promote oocyte release, lacks sufficient evidence to support its widespread clinical use in LUFS (16). In addition, a clinical study has shown that women with LUFS often experience lower pregnancy rates with IVF (17). Therefore, it is essential to explore appropriate and effective treatment strategies to improve the fertility outcomes in women with LUFS.

Acupuncture, a complementary and alternative intervention, has been widely used in the management of ovulatory dysfunction, with supporting evidence. A previous meta-analysis including 20 studies with 1,688 participants found that acupuncture significantly improved the pregnancy and ovulation rates and reduced the miscarriage rates in women with ovulatory disorder infertility (18). Leading organizations in the field, such as China Association of Chinese Medicine, recommend acupuncture for the treatment of ovulatory dysfunction (19). Recently, numerous randomized controlled trials (RCTs) have been conducted to investigate the efficacy of acupuncture in women with LUFS. However, some of these RCTs produced conflicting results. For instance, Zeng et al. (20) found that acupuncture intervention did not increase the ovulation rate compared with the control group treated with human chorionic gonadotropin (hCG). However, in 2025, Zhang et al. (21) reported dramatically enhanced ovulation rates after acupuncture therapy. Furthermore, Sun et al. (22) demonstrated that acupuncture treatment was associated with increased estradiol (E_2_) levels, a finding contrary to that of Tang et al. (23). These inconsistencies in previous RCT findings may have stemmed from methodological limitations, such as relatively small sample sizes and single-center designs, potentially limiting the robustness of their conclusions. Therefore, this meta-analysis specifically aimed to address the following question: Is acupuncture therapy effective for women with LUFS?

## 2 Materials and methods

This study (PROSPERO registration no. CRD420251062225) was conducted following the Preferred Reporting Items for Systematic Reviews and Meta-Analyses (PRISMA) guidelines (24).

### 2.1 Search strategy

We comprehensively searched eight databases, including PubMed, SinoMed, Scopus, Cochrane Library, Web of Science, China National Knowledge Infrastructure (CNKI), Wanfang, and VIP Information, from database inception to May 3, 2025. ClinicalTrials.gov, as an additional potential data source, was also searched. The search strategy was performed using the following three components: clinical condition (luteinized unruptured follicle syndrome, unruptured follicle syndrome, and LUFS); intervention (electroacupuncture and acupuncture); and study type (RCTs, randomized controlled trial). The full search strategy is provided in **Supplementary Material 1**. In addition, two investigators independently screened the titles, abstracts, and full texts to assess the eligibility of the articles. The reference lists of the retrieved articles were also manually searched to identify additional eligible studies. There were no geographical restrictions during the study search. Any disagreement was resolved through discussion with the third author, if necessary.

### 2.2 Inclusion and exclusion criteria

Studies were included if they met the following criteria: 1) included women diagnosed with LUFS based on recognized diagnostic criteria (25); 2) eligible interventions were acupuncture, including electroacupuncture and manual acupuncture, regardless of the needling techniques; 3) the study design is RCT evaluating the efficacy of acupuncture for LUFS; 4) to ensure comparability, only trials that allocated identical concomitant therapies (e.g., herbal medicine and letrozole) to both the intervention and control arms were included, with valid control comparators including pharmacological interventions, sham acupuncture, waitlist controls, routine care, and untreated groups; and 5) articles written in English or Chinese.

The exclusion criteria were as follows: 1) participants with reproductive tumors, intrauterine adhesion, diminished ovarian reserve, and chromosomal abnormalities; 2) the study intervention combined acupuncture with moxibustion or used acupoint catgut embedding; and 3) the study is a review, an animal experiment, a study protocol, a conference paper, a duplicate publication, or a meta-analysis.

### 2.3 Data extraction and risk of bias assessment

Two investigators independently extracted the relevant data using standardized forms, which included the author’s last name, the sample size, the publication year, the age of the participants, the treatment regimen, the number of acupoints, the treatment duration, and the outcomes. The primary outcomes were the ovulation rate and the pregnancy rate. The secondary outcomes were the luteinizing hormone (LH), E_2_, and progesterone levels, the resistance index (RI), and the pulsatility index (PI) of the ovarian artery. In addition, two investigators independently examined the quality of the studies according to the instructions in the Cochrane Handbook (http://handbook.cochrane.org). The included RCTs were assigned to low, high, or unclear risk of bias (RoB). Any disagreement was resolved by consulting with the third author, if necessary.

### 2.4 Statistical analysis

Review Manager 5.3 was used for meta-analysis, quality assessment, and subgroup analysis, whereas Stata 15.1 was utilized for publication bias analysis. For continuous outcomes (e.g., sex hormone levels), the results were summarized using the mean difference (MD) and standardized MD (SMD) with 95% confidence intervals (CIs). For dichotomous outcomes (e.g., the ovulation and pregnancy rates), the risk difference (RD) with 95%CIs was used. Heterogeneity among studies was assessed using the *I* ^2^ statistic. Significant heterogeneity was defined as *I* ^2^ > 50%. A random-effects model was used when significant heterogeneity was present; otherwise, a fixed-effects model was applied. Statistical significance was defined as *p* < 0.05 (26). When more than five trials were included, sensitivity analysis was conducted by systematically excluding each study individually to assess the robustness of the pooled results (27). Where possible and appropriate, a predefined subgroup analysis was performed based on the number of acupoints used per treatment (six or fewer acupoints or greater than six acupoints). Moreover, where >10 studies were available, publication bias was evaluated using Begg’s and Egger’s tests. A *p*-value >0.05 indicated the absence of publication bias.

## 3 Results

### 3.1 Included studies

**Figure 1:**
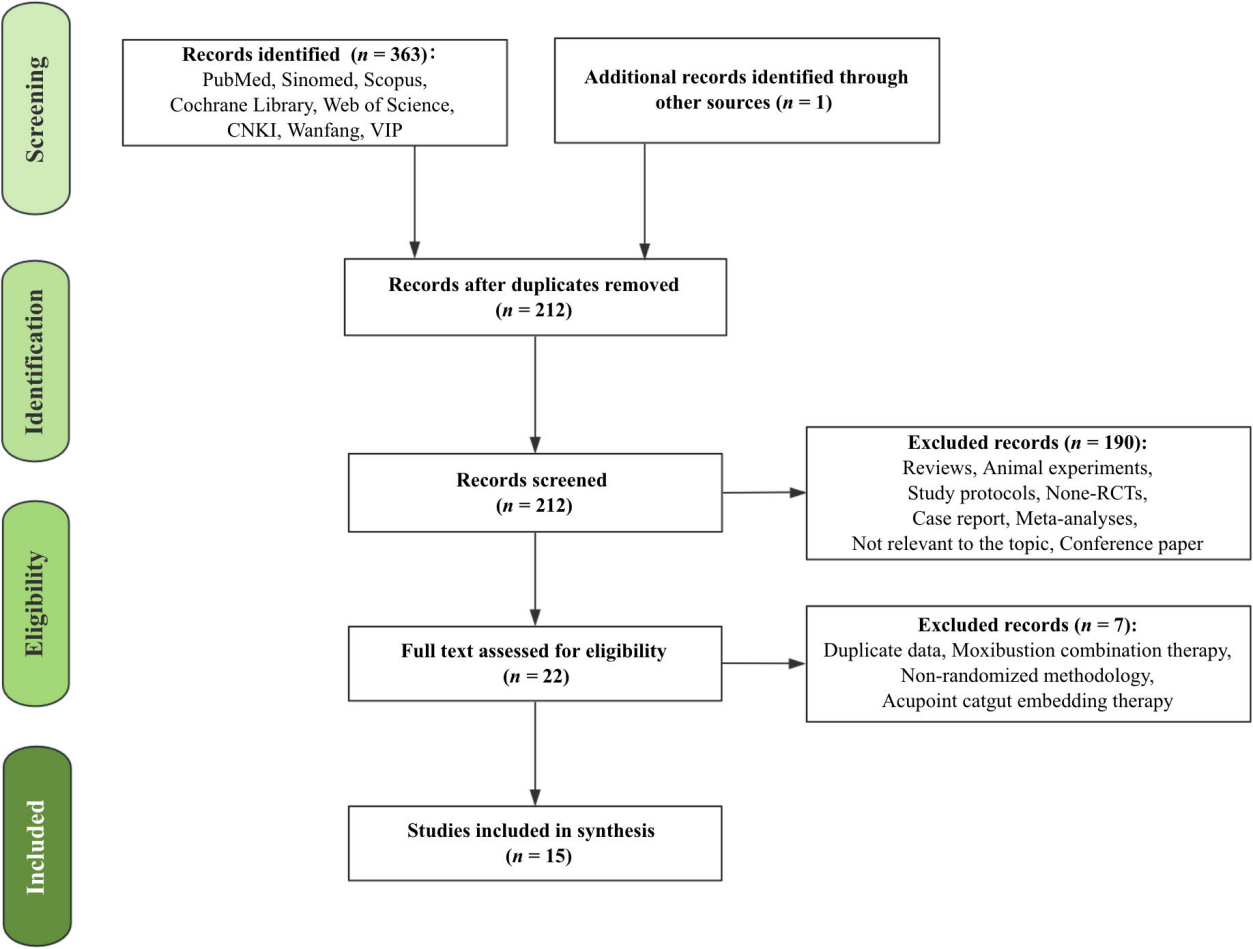
Flow diagram.

The study selection process is detailed in the PRISMA flow diagram (**Figure 1**), exhibiting the article selection procedure. According to the search strategy, a total of 363 records were identified through the screening of eight databases, and one additional record was identified from other sources (ClinicalTrials.gov). After removal of duplicates, 212 studies remained. Screening of the titles and abstracts resulted in the exclusion of 190 records based on the exclusion criteria. Full-text assessment of the remaining 22 articles led to the exclusion of seven studies for the following reasons: 1) duplicate data; 2) interventions combining acupuncture with moxibustion; 3) non-randomized study design; and 4) use of acupoint catgut embedding therapy. Ultimately, after the selection process, 15 records were retained for qualitative synthesis.

### 3.2 Study characteristics

**Table 1** shows information regarding the fundamental characteristics of all the included trials. This meta-analysis included 15 RCTs that involved a total of 1,030 participants. The sample size of individual trials ranged from 40 to 108. The trial and control groups included 503 and 535 participants, respectively. All 15 trials were conducted in China between 2005 and 2025. The participants’ ages in the trial and control groups were separately documented in 13 trials. Two studies used electroacupuncture, while 11 used manual acupuncture. In addition, the control comparisons were as follows: three studies compared the efficacy of acupuncture plus human chorionic gonadotropin (hCG) with hCG alone; three studies compared acupuncture with hCG; five studies compared acupuncture plus hCG plus Chinese herbal medicine (CHM) with hCG plus CHM; one study compared acupuncture plus CHM with CHM; one study compared acupuncture plus hCG plus CHM plus letrozole with hCG plus CHM plus letrozole; and two studies compared acupuncture plus hCG plus CC with hCG plus CC. The drug dosages are documented in **Supplementary Material 2**. The number of acupoints used per treatment varied from 4 to 26 acupoints. Furthermore, the treatment duration was reported in 11 RCTs and ranged from 4 weeks to three menstrual cycles.

**Table 1:** Study characteristics.

Study	Year	Sample size (*n*)		Participants’ age (years)		Treatment regimen		No. of acupoints	Treatment duration	Outcome
		Trial	Control	Trial	Control	Trial	Control			
Feng (21)	2025	46	46	29.42 ± 5.48	28.62 ± 4.67	MA + hCG + CC	hCG + CC	10	3 menstrual cycles	OR, PR, LH, E_2_, RI, PI
Li (28)	2022	36	36	29.6 ± 3.4		MA + hCG	hCG	8	4 weeks	PR, LH, E_2_, P, RI, PI
Zhang (29)	2021	30	30	31.25 ± 2.96	31.79 ± 3.62	MA + hCG	hCG	7	3 menstrual cycles	OR, PR, LH, E_2_, RI, PI
Zhang (30)	2021	20	20	27.9 ± 2.3	29.4 ± 2.4	MA + CHM	CHM	4	3 menstrual cycles	OR, RI, PI
Tang (31)	2019	30	30	30.13 ± 4.53	31.26 ± 3.87	MA + hCG	hCG	26	3 menstrual cycles	OR
Xu (32)	2018	32	33	30.15 ± 3.00	30.18 ± 3.07	MA + hCG + CHM + LE	hCG + CHM + LE	6	NA	OR, PR, RI, PI
Zhu (33)	2018	44	44	28. 21 ± 2. 25	27. 21 ± 3. 25	MA + hCG + CHM	hCG + CHM	9	3 menstrual cycles	OR, PR, LH, E_2_, RI, PI
Tang (23)	2017	30	30	29.07 ± 4.45	28.13 ± 3.17	MA + hCG + CHM	hCG + CHM	6	3 menstrual cycles	OR, PR, LH, E_2_, P
Zeng (20)	2017	32	76	27.85 ± 3.456	27.49 ± 3.022	EA	hCG	6	NA	OR, PR, E_2_, P
Xu (34)	2017	39	37	30.41 ± 3.55	30.16 ± 3.31	MA + hCG + CHM	hCG + CHM	6	NA	PR, LH, E_2_, P, RI, PI
Guo (35)	2017	30	30	33.23 ± 2.62	33.47 ± 2.21	MA	hCG	8	3 menstrual cycles	OR, PR, RI, PI
Wang (36)	2016	45	42	30.43 ± 3.42	30.30 ± 3.21	MA + hCG + CHM	hCG + CHM	6	3 menstrual cycles	OR, PR, LH, E_2_, P
Sun (22)	2015	31	30	30.80 ± 3.6	30.9 ± 3.6	MA + hCG + CHM	hCG + CHM	7	3 menstrual cycles	OR, PR, LH, E_2_, RI, PI
Liu (37)	2011	23	21	30.48 ± 4.03	29.71 ± 3.05	MA	hCG	7	3 menstrual cycles	OR, PR, RI, PI
Jin (38)	2005	35	30	27 ± 2.13		EA + hCG + CC	hCG + CC	7	NA	OR

*MA*, manual acupuncture; *EA*, electroacupuncture; *CHM*, Chinese herbal medicine; *hCG*, human chorionic gonadotropin; *LE*, letrozole; *CC*, clomiphene citrate; *NA*, not available; *OR*, ovulation rate; *PR*, pregnancy rate; *LH*, luteinizing hormone; *E_2_*, estradiol; *P*, progesterone; *RI*, resistance index; *PI*, pulsatility index.

### 3.3 Risk of bias assessment

**Figure 2:**
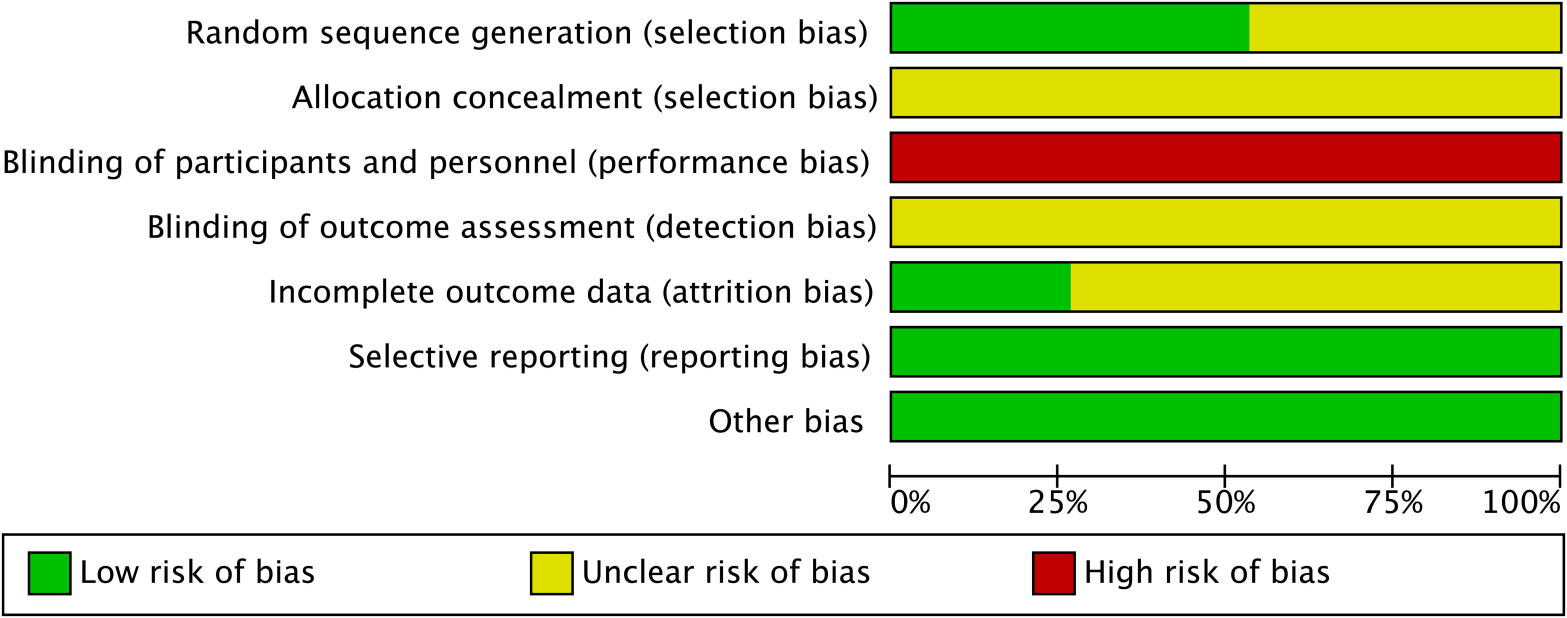
Risk of bias graph.

All of the included studies mentioned random sequence generation. However, 7 of the 15 trials (20–23, 29, 34, 36) were judged to have an unclear RoB as these trials only stated that assignment was “random” without describing the method used to generate the random sequence. All of the 15 studies provided inadequate information on allocation concealment and were therefore judged as having an unclear RoB. Due to the lack of reported blinding of participants and personnel, all of the included trials were judged to have a high risk of performance bias. Although blinding of outcome assessment was not implemented in any of these studies, they were still judged as having unclear RoB due to the observed outcomes (e.g., ovulation rate, pregnancy rate, and sex hormone levels) being objective measures and may not be affected by blinding procedures. Furthermore, incomplete outcome data (attrition bias) were adequately addressed in four studies (21, 30, 31, 37), which were therefore assessed as having a low RoB. There were no selective reporting outcomes and other bias detected in these 15 studies; thus, a low RoB was assessed (**Figure 2**). Detailed RoB assessment for each study is provided in **Supplementary Material 3**.

### 3.4 Outcome measurements

#### 3.4.1 Primary outcomes

**Figure 3:**
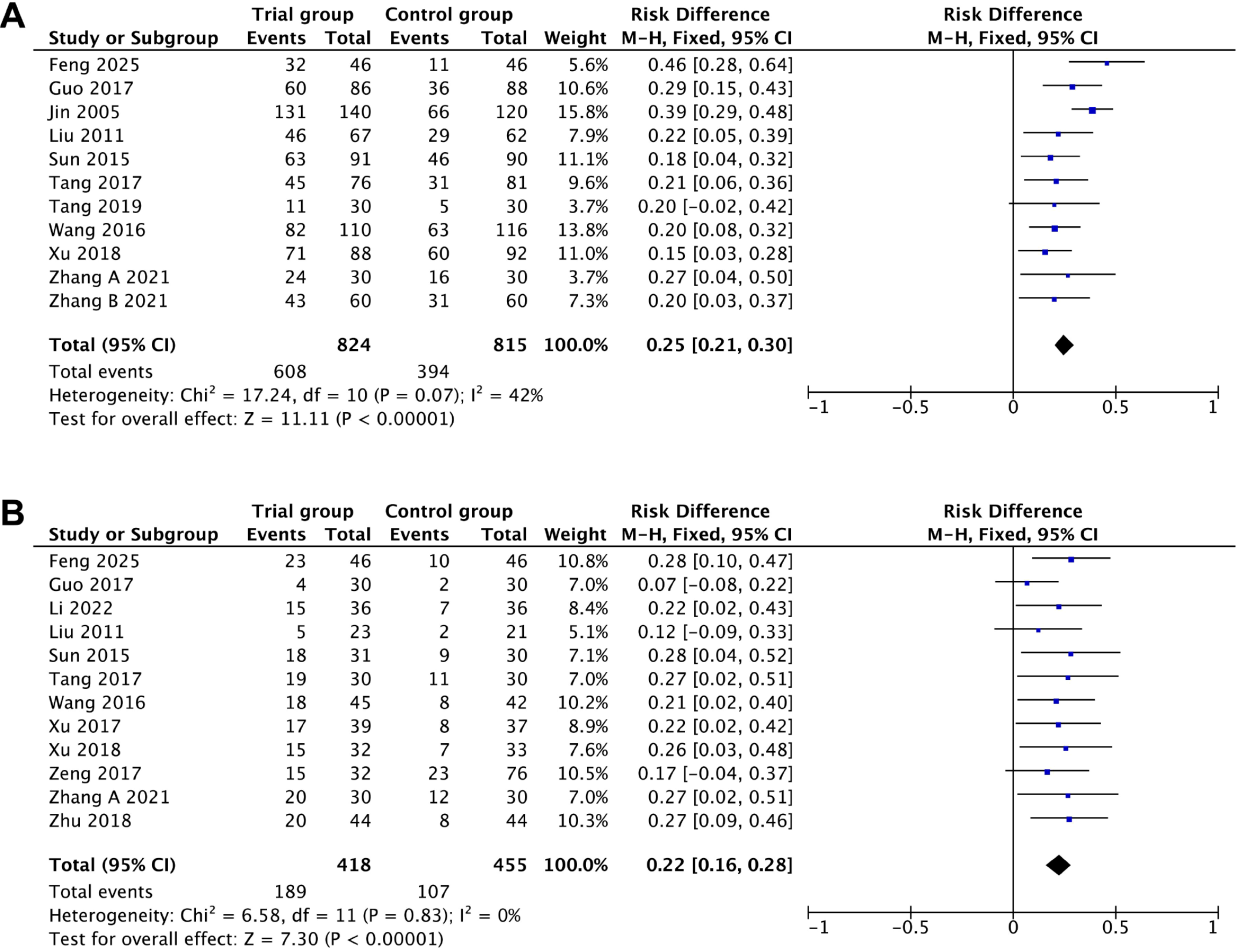
Primary outcomes. **(A)** Ovulation rate. **(B)** Pregnancy rate.

A total of 12 studies, encompassing 2,012 treatment cycles, contributed data on the association between acupuncture intervention and ovulation rate. After removing one trial (20) through sensitivity analysis, the heterogeneity across studies decreased from 70% to 42%, and the findings suggest that acupuncture significantly increased the ovulation rate by 25% (RD = 0.25, 95%CI = 0.21–0.30, *p* < 0.00001, *I* ^2^ = 42%) (**Figure 3A**, **Table 2**).

**Table 2:** Results of the forest plots for the clinical outcomes.

Clinical outcomes	Studies (*n*)	Cases (*n*)	RD/SMD/MD (95%CI)	*p*	*I* ^2^ (%)	Model
Primary outcomes						
Ovulation rate	11	1,639	0.25 (0.21–0.30)	<0.00001	42	Fixed
Pregnancy rate	12	873	0.22 (0.16–0.28)	<0.00001	0	Fixed
Secondary outcomes						
Luteinizing hormone	7	536	3.76 (2.27–5.25)	<0.00001	78	Random
Estradiol	8	632	0.47 (0.31–0.63)	<0.00001	12	Fixed
Progesterone	5	431	1.50 (1.09–1.91)	<0.00001	50	Fixed
Resistance index	10	658	−0.07 (−0.09 to −0.05)	<0.00001	59	Random
Pulsatility index	10	658	−0.10 (−0.15 to −0.06)	<0.00001	74	Random
Subgroup analysis						
Ovulation rate (≤6 acupoints)	4	683	0.19 (0.12–0.26)	<0.00001	0	Fixed
Ovulation rate (>6 acupoints)	8	1,180	0.28 (0.23–0.33)	<0.00001	41	Fixed
Pregnancy rate (≤6 acupoints)	5	396	0.22 (0.12–0.31)	<0.00001	0	Fixed
Pregnancy rate (>6 acupoints)	7	477	0.23 (0.15–0.31)	<0.00001	3	Fixed

Regarding the pregnancy rate, the pooled results from 12 studies that included 873 participants demonstrated a 22% higher pregnancy rate in the acupuncture group compared with the control group (RD = 0.22, 95%CI = 0.16–0.28, *p* < 0.00001, *I* ^2^ = 0%) (**Figure 3B**, **Table 2**). The sensitivity analysis confirmed the robustness of the results.

#### 3.4.2 Secondary outcomes

**Figure 4:**
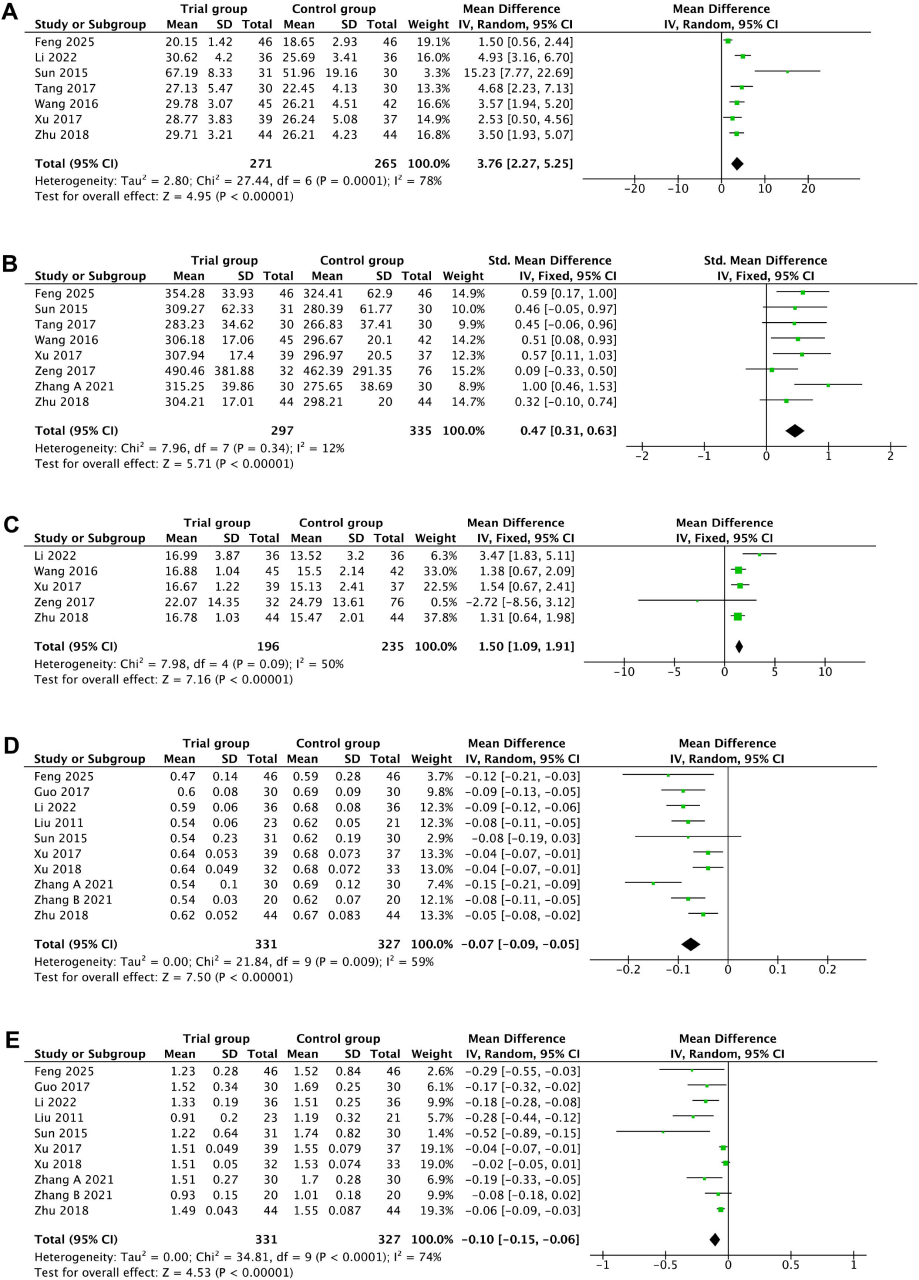
Secondary outcomes. **(A)** Luteinizing hormone. **(B)** Estradiol. **(C)** Progesterone. **(D)** Resistance index. **(E)** Pulsatility index.

Eight trials assessed the LH levels according to whether or not the participants received acupuncture intervention. After excluding one study (29) through sensitivity analysis, the heterogeneity declined from 98% to 78%; thus, there was evidence that the LH levels significantly increased when acupuncture was administered (MD = 3.76, 95%CI = 2.27–5.25, *p* < 0.00001, *I* ^2^ = 78%) (**Figure 4A**, **Table 2**).

Nine studies reported the E_2_ levels in women with acupuncture intervention. One study (29) was removed after sensitivity analysis, with the heterogeneity decreasing from 44% to 12%. The pooled SMD was 0.47 (95%CI = 0.31–0.63, *p* < 0.00001, *I* ^2^ = 12%), revealing that acupuncture remarkably improved the E_2_ levels in women with LUFS (**Figure 4B**, **Table 2**).

The progesterone levels were reported in six trials. Following the removal of one study (23) in the sensitivity analysis, the heterogeneity decreased significantly from 82% to 58%. The pooled data from this study showed that acupuncture may increase the progesterone levels (MD = 1.50, 95%CI = 1.09–1.91, *p* < 0.00001, *I* ^2^ = 50%) (**Figure 4C**, **Table 2**).

Simultaneously, statistically significant differences were detected in both the RI (MD = −0.07, 95%CI = −0.09 to −0.05, *p* < 0.00001, *I* ^2^ = 59%) and PI (MD = −0.10, 95%CI = −0.15 to −0.06, *p* < 0.00001, *I* ^2^ = 74%) of the ovarian artery between the acupuncture group and the control group (**Figures 4D, E**, **Table 2**). The sensitivity analysis indicated that no single trial significantly affected the pooled estimates.

#### 3.4.3 Adverse events

Safety represents a paramount consideration in clinical trials. Although adverse events (AEs) were mentioned in 6 of the 15 included studies (23, 28, 31, 32, 34, 37), only one (28) provided comparative intergroup data. Four studies (23, 32, 34, 37) reported the absence of serious AEs during treatment. Mild nausea, vomiting, and bruising occurred in the acupuncture groups of two studies (28, 31). Collectively, these findings demonstrate a favorable safety profile for acupuncture in the management of LUFS.

#### 3.4.4 Subgroup analysis

**Figure 5:**
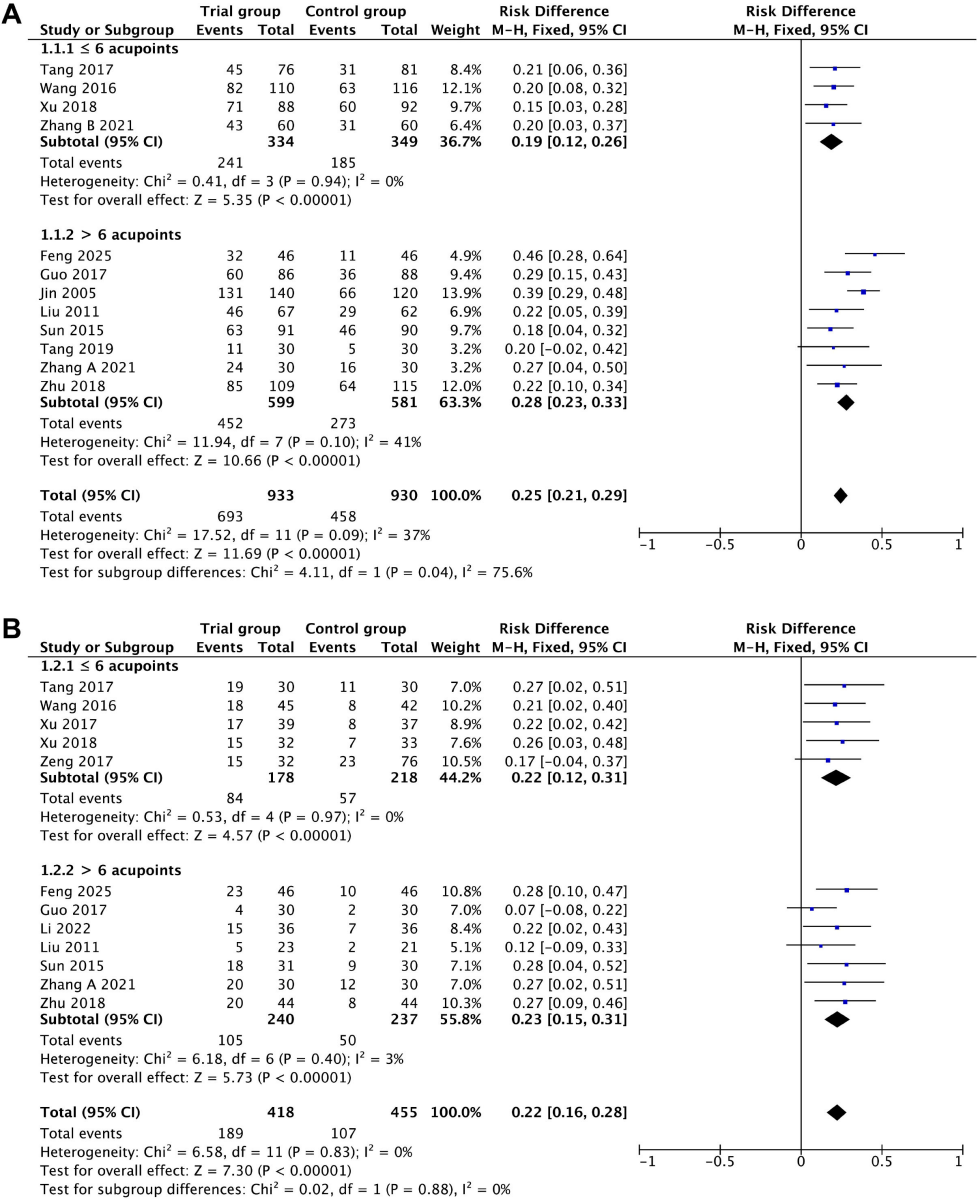
Subgroup analysis of the dose–response relationships. **(A)** Ovulation rate. **(B)** Pregnancy rate.

Subgroup analysis was conducted to explore the potential influence of the number of acupoints used per treatment (six or fewer acupoints *vs*. more than six acupoints) on the ovulation and pregnancy rates. Stimulation of more than six acupoints was associated with a significantly higher increase in ovulation rate (28%, RD = 0.28, 95%CI 0.23 = 0.33, *p* < 0.00001, *I* ^2^ = 41%) compared with stimulation of six or fewer acupoints (19%; RD = 0.19, 95%CI = 0.12 0.26, *p* < 0.00001, *I* ^2^ = 0%) per treatment course (**Figure 5A**, **Table 2**).

In contrast, for the pregnancy rate, there was no significant difference between stimulation of six or fewer acupoints (22%; RD = 0.22, 95%CI = 0.12–0.31, *p* < 0.00001, *I* ^2^ = 0%) and stimulation of more than six acupoints (23%; RD = 0.23, 95%CI = 0.15–0.31, *p* < 0.00001, *I* ^2^ = 3%) (**Figure 5B**, **Table 2**).

### 3.5 Publication bias

**Figure 6:**
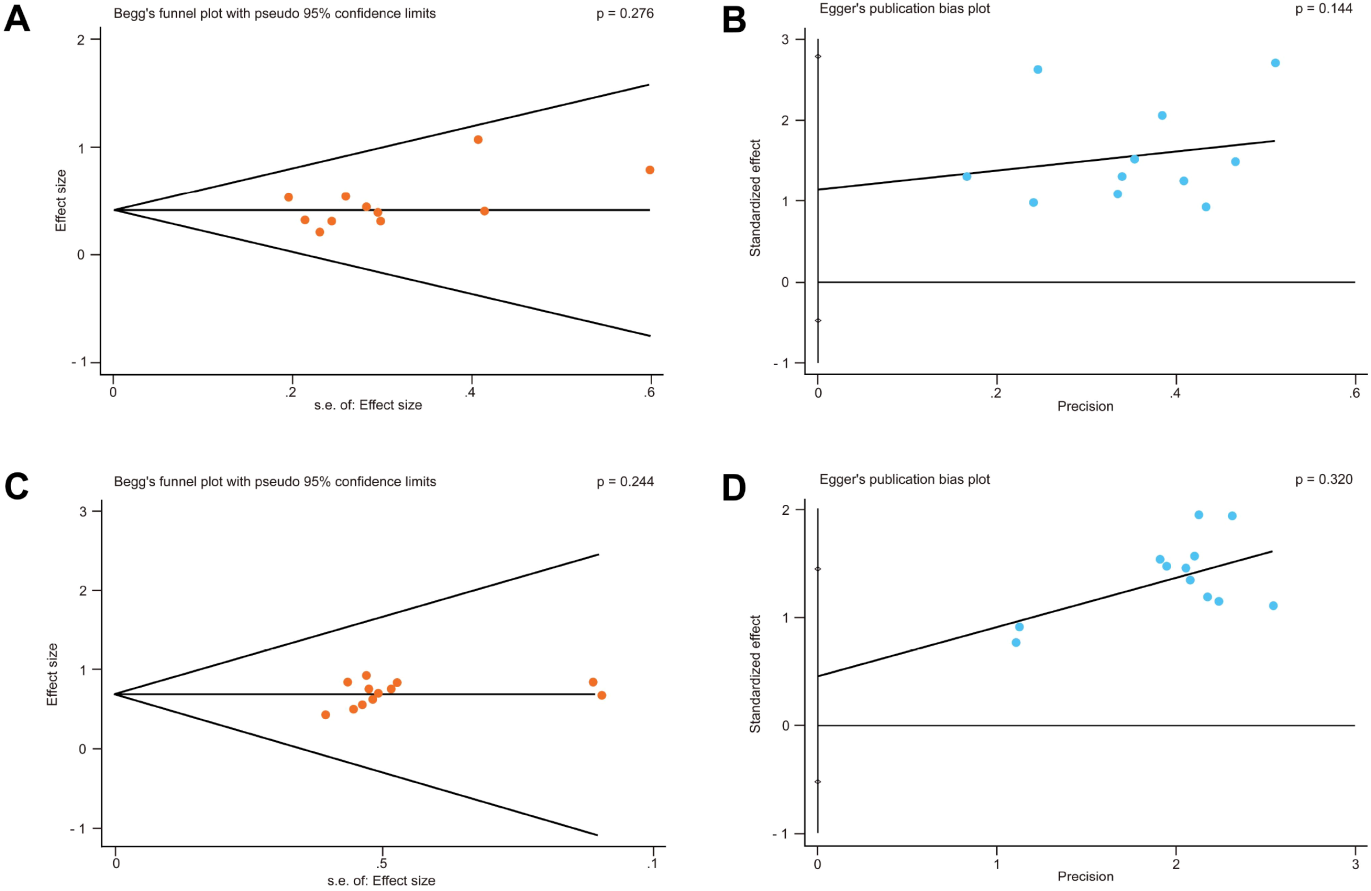
Publication bias analysis. Begg’s **(A)** and Egger’s **(B)** tests for the ovulation rate. Begg’s **(C)** and Egger’s **(D)** tests for the pregnancy rate.

Publication bias constitutes a critical validity issue in systematic reviews by distorting the evidence pools and meta-analysis estimates (39). Hence, potential publication bias was assessed using Begg’s and Egger’s tests. For the ovulation rate outcome, the *p*-values from the Begg’s and Egger’s tests were 0.276 and 0.144, respectively (**Figures 6A, B**), suggesting no significant publication bias. Similarly, for the pregnancy rate, the results of the Begg’s test (*p* = 0.244) and the Egger’s test (*p* = 0.320) indicated no significant publication bias (**Figures 6C, D**).

## 4 Discussion

Current guidelines lack recommendations for LUFS interventions. Therefore, numerous clinicians have been exploring novel approaches to improve the fertility outcomes in women with LUFS. Acupuncture, a promising non-pharmacological treatment in the reproductive field, has been clarified to comprise various mechanisms. Mounting studies have indicated that the autophagy of ovarian granulosa cells constitutes a major cause of abnormal follicular development and ovulation dysfunction. However, acupuncture intervention may improve this condition by suppressing LncMEG3 expression, thereby inhibiting the PI3K/Akt/mTOR signaling pathway (40). Furthermore, acupuncture may improve ovulatory dysfunction by inhibiting apoptosis of ovarian granulosa cells through targeting miR-21-3p (41). Moreover, acupuncture treatment has been associated with the regulation of the theca interna cell layer, the antral follicles, follicle-stimulating hormone (FSH) receptor mRNA expression in the ovary, and the circulating estrogen concentrations (42). Interestingly, an experimental study on 11 female donkeys reported a higher ovulation rate in the acupuncture group (72.73%) compared with the hCG group (18.18%). This was accompanied by increased serum progesterone concentrations and number of colored pixel as measured by color Doppler ultrasound (US), demonstrating the potential efficacy of acupuncture for inducing ovulation (43). Moreover, ovarian innervation plays a vital role in promoting folliculogenesis and ovulation. Thus, Tong et al. suggested that acupuncture might restore ovulation by mediating the superior ovarian nerve (44). Beta-nerve growth factor (β-NGF) critically regulates the neuroendocrine and reproductive system. Substantial evidence demonstrates that β-NGF can promote the differentiation of follicular cells to luteal cells, induce the release of gonadotropin-releasing hormone (GnRH) and LH, and trigger ovulation (45, 46). Notably, acupuncture may promote ovulation by normalizing the sympathetic ovarian response to NGF action (47). A previous study focusing on the connection of acupuncture and the hypothalamic–pituitary–gonadal axis suggested that acupuncture may decrease the proportion of atretic follicles by enhancing the pituitary ERβ expression (48). Furthermore, clinical studies have provided insights into the mechanisms of acupuncture for ovulatory dysfunction, revealing its potential to induce ovulation by modulating the cortisol and sex hormone levels, including estrone, estrone sulfate, androsterone glucuronide, and free testosterone (49, 50).

### 4.1 Main results

This meta-analysis provided evidence that acupuncture is beneficial for improving the ovulation and pregnancy rates, as well as modifying the sex hormones levels including LH, E_2_, and progesterone, along with the RI and PI of the ovarian artery. Subgroup analysis suggested that stimulation of more than six acupoints was associated with a higher ovulation rate increase (28%) compared with stimulation of six or fewer acupoints (19%). In contrast, the dose of acupoints stimulated showed no significant association with the pregnancy rate increase (22% *vs*. 23%). Furthermore, the sensitivity analysis showed the results to be robust and not driven by any single study. In addition, the absence of publication bias, as confirmed by the Begg’s and Egger’s tests, indicated the reliability of the findings. Clinically, although infertile women with LUFS often pursue ART, challenges such as suboptimal ovulation induction outcomes, high LUFS recurrence rates, and low pregnancy rates pose therapeutic dilemmas (13). Nevertheless, this meta-analysis highlighted the potential value of acupuncture in promoting the ovulation and pregnancy rates in this population. Furthermore, it is well established that LH and estrogen play fundamental roles in the ovulatory cycle, and evidence suggests that low levels of these hormones may contribute to poor ovulation rates (51). Women with LUFS often exhibit elevated ovarian artery RI and PI, which are well-recognized indices inversely correlated with ovulatory function (52). This meta-analysis revealed that the LH and estrogen levels and the RI and PI could be considerably improved after intervention with acupuncture. Interestingly, our subgroup analysis suggested that stimulating a higher number of acupoints (more than six) may be associated with greater improvements in the ovulation rate. This implied that using more acupoints could be considered in clinical practice to optimize the ovulation outcomes in women with LUFS. Moreover, although the reporting of AEs is a key clinical concern, only six (23, 28, 31, 32, 34, 37) of the 15 RCTs explicitly documented AEs. Among them, four studies (23, 32, 34, 37) reported no AEs (e.g., ovarian hyperstimulation syndrome), while the other two (28, 31) noted only mild AEs (i.e., bruising, nausea, and vomiting) in the acupuncture group. Consequently, quantitative analysis of the AEs was not feasible due to insufficient reported data.

### 4.2 Differences with other studies

A comparable meta-analysis (53) of women with LUFS in 2020 showed the benefits of acupuncture on the ovulation rates, the LH and E_2_ levels, and the RI and PI, but no improvement in the pregnancy rates. Our meta-analysis differs from this prior work in several key aspects: firstly, the previous study lacked a registered protocol and searched only six databases (up to July 2019), including 10 studies with 715 participants. In contrast, our meta-analysis followed a pre-registered protocol, comprehensively searched eight databases and one clinical trial database, and included 15 RCTs involving 1,030 women with LUFS. In addition, this meta-analysis documents comprehensive search strategies for each database, enhancing the methodological reproducibility relative to previous reviews. Secondly, while the previous meta-analysis pooled the results for six outcomes from 178 sample sizes and found insufficient evidence for an improvement in the pregnancy rate (*p* = 0.08), our analysis evaluated seven outcomes and demonstrated a significant improvement in the pregnancy rate based on data from 873 participants. We also adopted the RD statistical method to present the results in this study. For instance, the acupuncture intervention increased the ovulation rate by 25% and the pregnancy rate by 22%, which may offer a more intuitive understanding of its clinical impact. Thirdly, to our knowledge, this is the first meta-analysis to investigate the influence of the number of acupoints used per treatment on the primary outcomes. Our findings suggest that stimulation of more than six acupoints may yield a greater improvement in the ovulation rate (28%) than stimulation of six or fewer acupoints (19%), while the dose of acupoints showed no significant association with the pregnancy rate increase (22% *vs*. 23%). Lastly, we performed sensitivity analyses and the Begg’s and Egger’s tests for publication bias, which were not reported in the previous meta-analysis, strengthening the robustness and reliability of our findings. Therefore, the findings of this meta-analysis may motivate further research into the clinical value of acupuncture for women with LUFS.

### 4.3 Limitations of this research

Nevertheless, several limitations warrant consideration. Firstly, while high-quality trials on acupuncture for ovulatory dysfunction exist outside China, e.g., in the US (54) and Sweden (55), only RCTs conducted in China met our inclusion criteria for this specific LUFS meta-analysis. Consequently, the generalizability of our findings to non-Chinese populations may be limited. Secondly, although our findings suggest that acupuncture may benefit LUFS outcomes, it is important to note that LUFS likely has multiple etiological pathways. We were unable to perform subgroup analyses based on the underlying causes of LUFS. Hence, it remains unknown whether acupuncture exerts different therapeutic effects on LUFS resulting from distinct etiologies. Thirdly, the methodological reporting in many of the included studies was suboptimal, which made the evidence quality moderate to low due to methodological concerns. For instance, seven studies merely described allocation as “random” without detailing the method, and no studies adequately reported blinding procedures. As a result, this limitation may attenuate the strength of our conclusions. Nevertheless, evidence from open-label studies indicates an inherent RoB due to non-blinding, and non-blinded pragmatic trials have gained increasing endorsement in recent years for generating clinically relevant outcomes. This preference stems from their emphasis on real-world extrapolation and practical applicability (enhancing the external validity) rather than solely focusing on treatment efficacy (56). Such trial designs are particularly well suited for the evaluation of complex, flexible interventions such as acupuncture (57). Finally, AE reporting was insufficient. Only one study (28) provided detailed AE rates of two groups. Therefore, quantitative synthesis of AEs was precluded, limiting our assessment of the safety profile of acupuncture in this context.

### 4.4 Implications for future research

Firstly, the efficacy and the safety of acupuncture therapy are primary concerns in clinical practice. None of the included studies reported protocol registration in established trial registries (e.g., Chinese Clinical Trial Registry); thereby, the majority likely failed to conduct comprehensive safety assessments for the clinical trials. Moreover, 15 studies failed to predefine AEs, which may have contributed to the underreporting of safety outcomes. Future RCTs should prioritize standardized reporting of AEs according to guidelines such as CONSORT (58) in order to better characterize the safety profile of acupuncture for LUFS, despite the suggestion of no serious AEs in the included studies. Secondly, employing rigorous controls, such as sham acupuncture and blinding, is crucial in future trials to provide more robust evidence on the specific efficacy of acupuncture. This would significantly strengthen the conclusions drawn from such studies. Thirdly, research exploring whether the effectiveness of acupuncture varies based on the underlying etiology of LUFS is also needed. This would strengthen the current evidence base. Fourthly, despite a considerable number of studies reporting the mechanisms of acupuncture for ovulation disorders, studies directly investigating the mechanisms of acupuncture for LUFS are relatively scarce. Therefore, conducting dedicated mechanistic research such as biochemical and imaging studies to elucidate the specific mechanisms of acupuncture for LUFS is imperative in future studies. Lastly, the association between improved ovulation rates and the use of more than six acupoints, derived indirectly from subgroup analyses, is suggestive, but not conclusive. Future trials should validate this exploratory finding by stratifying participants into cohorts that either had more than six acupoints or had six or fewer acupoints to assess potential dose–response relationships.

## 5 Conclusion

The current evidence from Chinese RCTs suggests that, in women with LUFS, acupuncture intervention increases the ovulation rate by 25% and the pregnancy rate by 22%. Interestingly, this meta-analysis provided evidence that stimulation of more than six acupoints may be associated with a greater improvement in the ovulation rate compared with stimulation of six or fewer acupoints. However, further rigorously designed multi-country trials and sham-controlled RCTs are needed to confirm these findings and to establish the clinical value of acupuncture for LUFS.

## Data Availability

The original contributions presented in the study are included in the article/**Supplementary Material**. Further inquiries can be directed to the corresponding author.

## References

[1] Boivin J Bunting L Collins JA Nygren KG . International estimates of infertility prevalence and treatment-seeking: potential need and demand for infertility medical care. Hum Reprod. (2007) 22:1506–12. doi: 10.1093/humrep/dem046, PMID: 17376819

[2] Liang Y Huang J Zhao Q Mo H Su Z Feng S . Global, regional, and national prevalence and trends of infertility among individuals of reproductive age (15 – 49 years) from 1990 to 2021, with projections to 2040. Hum Reprod. (2025) 40:529–44. doi: 10.1093/humrep/deae292, PMID: 39752330

[3] Carson SA Kallen AN . Diagnosis and management of infertility: A review. JAMA. (2021) 326:65–76. doi: 10.1001/jama.2021.4788, PMID: 34228062 PMC9302705

[4] Zhou Z Zheng D Wu H Li R Xu S Kang Y . Epidemiology of infertility in China: a population-based study. BJOG. (2018) 125:432–41. doi: 10.1111/1471-0528.14966, PMID: 29030908

[5] Bell SO Moreau C Sarnak D Kibira SPS Anglewicz P Gichangi P . Measuring non-events: infertility estimation using cross-sectional, population-based data from four countries in sub-Saharan Africa. Hum Reprod. (2024) 39:2848–60. doi: 10.1093/humrep/deae218, PMID: 39348340 PMC11629970

[6] Bourrion B Panjo H Bithorel PL de la Rochebrochard E François M Pelletier-Fleury N . The economic burden of infertility treatment and distribution of expenditures overtime in France: a self-controlled pre-post study. BMC Health Serv Res. (2022) 22:512. doi: 10.1186/s12913-022-07725-9, PMID: 35428284 PMC9013027

[7] Lai JD Fantus RJ Cohen AJ Wan V Hudnall MT Pham M . Unmet financial burden of infertility care and the impact of state insurance mandates in the United States: analysis from a popular crowdfunding platform. Fertil Steril. (2021) 116:1119–25. doi: 10.1016/j.fertnstert.2021.05.111, PMID: 34246467

[8] The World Health Organization . Sexual and reproductive health: infertility terminology and definitions. (2015).

[9] Macaluso M Wright-Schnapp TJ Chandra A Johnson R Satterwhite CL Pulver A . A public health focus on infertility prevention, detection, and management. Fertil Steril. (2010) 93:16.e1–10. doi: 10.1016/j.fertnstert.2008.09.046, PMID: 18992879

[10] Morshed-Behbahani B Lamyian M Joulaei H Rashidi BH Montazeri A . Infertility policy analysis: a comparative study of selected lower middle- middle- and high-income countries. Global Health. (2020) 16:104. doi: 10.1186/s12992-020-00617-9, PMID: 33097089 PMC7583186

[11] Munro MG Balen AH Cho S Critchley HOD Díaz I Ferriani R . The FIGO ovulatory disorders classification system†. Hum Reprod. (2022) 37:2446–64. doi: 10.1093/humrep/deac180, PMID: 35984284 PMC9527465

[12] Qublan H Amarin Z Nawasreh M Diab F Malkawi S Al-Ahmad N . Luteinized unruptured follicle syndrome: incidence and recurrence rate in infertile women with unexplained infertility undergoing intrauterine insemination. Hum Reprod. (2006) 21:2110–3. doi: 10.1093/humrep/del113, PMID: 16613885

[13] Etrusco A Buzzaccarini G Cucinella G Agrusa A Di Buono G Noventa M . Luteinised unruptured follicle syndrome: pathophysiological background and new target therapy in assisted reproductive treatments. J Obstet Gynaecol. (2022) 42:3424–8. doi: 10.1080/01443615.2022.2153297, PMID: 36469701

[14] Wang R Kim BV van Wely M Johnson NP Costello MF Zhang H . Treatment strategies for women with WHO group II anovulation: systematic review and network meta-analysis. BMJ. (2017) 356:j138. doi: 10.1136/bmj.j138, PMID: 28143834 PMC5421445

[15] Kamath MS Maheshwari A Bhattacharya S Lor KY Gibreel A . Oral medications including clomiphene citrate or aromatase inhibitors with gonadotropins for controlled ovarian stimulation in women undergoing *in vitro* fertilisation. Cochrane Database Syst Rev. (2017) 11:CD008528. doi: 10.1002/14651858.CD008528.pub3, PMID: 29096046 PMC6486039

[16] Check JH Vaniver J Senft D DiAntonio G Summers D . The use of granulocyte colony stimulating factor to enhance oocyte release in women with the luteinized unruptured follicle syndrome. Clin Exp Obstet Gynecol. (2016) 43:178–80. doi: 10.12891/ceog3229.2016, PMID: 27132403

[17] Demyttenaere K Nijs P Evers-Kiebooms G Koninckx PR . Personality characteristics, psychoendocrinological stress and outcome of IVF depend upon the etiology of infertility. Gynecol Endocrinol. (1994) 8:233–40. doi: 10.3109/09513599409023626, PMID: 7709762

[18] Chen YQ Shen T Lv Y Shen MH . Feasibility of acupuncture as an adjunct intervention for ovulatory disorder infertility: A systematic review and meta-analysis. World J Clin cases. (2024) 12:5108–23. doi: 10.12998/wjcc.v12.i22.5108, PMID: 39109015 PMC11238799

[19] China Association of Chinese Medicine . Clinical practice guidelines for integrated traditional Chinese and Western Medicine in the management of ovulatory disorder infertility. J Traditional Chin Med. (2024) 65:976–84. doi: 10.13288/j.11-2166/r.2024.09.019

[20] Zeng B . Clinical study on traditional Chinese medicine for infertility due to luteinized unruptured follicle syndrome. Nanjing Univ Chin Med. (2017).

[21] Feng Y Liu X Li B Li C Dou N . Efficacy of acupuncture combined with human chorionic gonadotropin in treatment of infertility with unruptured follicle luteinization syndrome. J Hubei Univ Chin Med. (2025) 27:95–7. doi: 10.3969/j.issn.1008-987x.2025.01.26

[22] Sun J Li J Zhang X . Clinical study on treatment of luteinized unruptured follicle syndrome with combination of TCM and Western medicine. China J Chin Med. (2015) 30:1804–7. doi: 10.16368/j.issn.1674-8999.2015.12.624

[23] Tang M . Clinical study on bushen cu pailuan tang combined with acupuncture in treating luteinized unruptured follicle syndrome with kidney deficiency and blood stasis pattern. Nanjing Univ Chin Med. (2017).

[24] Page MJ McKenzie JE Bossuyt PM Boutron I Hoffmann TC Mulrow CD . The PRISMA 2020 statement: an updated guideline for reporting systematic reviews. BMJ. (2021) 372:n71. doi: 10.1136/bmj.n71, PMID: 33782057 PMC8005924

[25] Shibata T Makinoda S Waseda T Tomizawa H Fujii R Utsunomiya T . Granulocyte colony-stimulating factor as a potential inducer of ovulation in infertile women with luteinized unruptured follicle syndrome. Transl Res. (2016) 171:63–70. doi: 10.1016/j.trsl.2015.10.003, PMID: 26518992

[26] Lin G Yie SLJ Guo S Li X Xu L . Clinical evidence of acupuncture for amnestic mild cognitive impairment: A systematic review and meta-analysis of randomized controlled trials. Complement Ther Med. (2025) 88:103114. doi: 10.1016/j.ctim.2024.103114, PMID: 39617303

[27] Lin G Zhong X Li S Liu X Xu L . The clinical value of progestin-primed ovarian stimulation protocol for women with diminished ovarian reserve undergoing IVF/ICSI: a systematic review and meta-analysis. Front Endocrinol (Lausanne). (2023) 14:1232935. doi: 10.3389/fendo.2023.1232935, PMID: 37670890 PMC10476097

[28] Li Li Wang Y Liu H . Clinical observation on menstruation-regulating and fertility-promoting acupuncture for the treatment of luteinized unruptured follicle syndrome. Guangming J Chin Med. (2022) 37:1829–32. doi: 10.3969/j.issn.1003-8914.2022.10.043

[29] Zhang C Wang H Bi Y Chen G Gao X . Effect of BO’s abdominal acupuncture combined with HCG on serum E2,LH,FSH and ovulation rate in patients with LUFS of kidney-deficiency and blood stasis pattern. J Clin Acupuncture Moxibustion. (2021) 37:21–5. doi: 10.19917/j.cnki.1005-0779.021176

[30] Zhang Q Chen D Wang Q Zhang X Xing Yu Zhaoxia W . Acupuncture combined Bushen Tiaozhou Method treatment the clinical effect for luteinized unruptured follicle syndrome of kidney deficiency during ovulation time. Hebei J Traditional Chin Med. (2021) 43:1261–5. doi: 10.3969/j.issn.1002-2619.2021.08.007

[31] Tang Z . Clinical efficacy observation of zhuang medicine acupuncture in treating luteinized unruptured follicle syndrome. Guangxi University of Chinese Medicine (2019).

[32] Xu W Wang L Zhu X Tang M . Clinical study on acupuncture combined with medicine in treating luteinized unruptured follicle syndrome of patients with polycystic ovary syndrome after ovulation induction. J Chengdu Univ Traditional Chin Med. (2018) 41:37–9. doi: 10.13593/j.cnki.51-1501/r.2018.04.037

[33] Zhu X Guo H Xu W Huang L . Application of bushen ovulation decoction combined with acupuncture to control ovarian hemodynamics in the treatment of unruptured follicular luteinized syndrome. J Sichuan Traditional Chin Med. (2018) 36:159–62.

[34] Xu W . Effect of acupuncture combined with medicine in treating luteinized unruptured follicle syndrome by improving the ovarian blood flow parameters. J Chengdu Univ Traditional Chin Med. (2017) 40:29–32. doi: 10.13593/j.cnki.51-1501/r.2017.01.029

[35] Guo Y Wu Y Wei Y Lu Z Dai S . Clinical research of applying acupuncture method of ren and du meridians for adjustment and smoothing in treating luteinized unruptured follicle syndrome. J Sichuan Traditional Chin Med. (2017) 35:184–6.

[36] Wang L Xu W . Clinical research on acupuncture combined with medicine on luteinized unruptured folicle syndrome. J Nanjing Univ Traditional Chin Med. (2016) 32:32–4. doi: 10.14148/j.issn.1672-0482.2016.0032

[37] Liu L . Clinical observation of Bo’s abdominal acupuncture for ovulation induction therapy in luteinized unruptured follicle syndrome. Guangzhou Univ Chin Med. (2011).

[38] Jin L Wang X . Clinical observation of electroacupuncture combined with pharmacotherapy for luteinized unruptured follicle syndrome. Pract Clin J Integrated Traditional Chin Western Med. (2005) 5:26–7. doi: 10.3969/j.issn.1671-4040.2005.05.018

[39] Bartoš F Maier M Wagenmakers E-J Doucouliagos H Stanley TD . Robust Bayesian meta-analysis: Model-averaging across complementary publication bias adjustment methods. Res Synth Methods. (2023) 14:99–116. doi: 10.1002/jrsm.1594, PMID: 35869696 PMC10087723

[40] Chen X Tang H Liang Y Wu P Xie L Ding Y . Acupuncture regulates the autophagy of ovarian granulosa cells in polycystic ovarian syndrome ovulation disorder by inhibiting the PI3K/AKT/mTOR pathway through LncMEG3. BioMed Pharmacother. (2021) 144:112288. doi: 10.1016/j.biopha.2021.112288, PMID: 34653763

[41] Chen X He H Long B Wei B Yang P Huang X . Acupuncture regulates the apoptosis of ovarian granulosa cells in polycystic ovarian syndrome-related abnormal follicular development through LncMEG3-mediated inhibition of miR-21-3p. Biol Res. (2023) 56:31. doi: 10.1186/s40659-023-00441-6, PMID: 37303036 PMC10258959

[42] Stener-Victorin E Wu X . Effects and mechanisms of acupuncture in the reproductive system. Auton Neurosci. (2010) 157:46–51. doi: 10.1016/j.autneu.2010.03.006, PMID: 20350839

[43] Ribeiro MO Bittencourt RF Feliciano MAR Santana ALA Silva MAA Felix MD . Subdose of human chorionic gonadotropin applied at the Hou Hai acupoint on follicular dynamics and luteal development in donkeys. Anim Reprod. (2020) 17:e20200554. doi: 10.1590/1984-3143-AR2020-0554, PMID: 33791033 PMC7995259

[44] Tong X Liu Y Xu X Shi J Hu W Ma T . Ovarian innervation coupling with vascularity: the role of electro-acupuncture in follicular maturation in a rat model of polycystic ovary syndrome. Front Physiol. (2020) 11:474. doi: 10.3389/fphys.2020.00474, PMID: 32547407 PMC7273926

[45] Carrasco RA Pezo S Zwiefelhofer EM Lanigan EE Singh J Berland MA . Is seminal nerve growth factor-induced luteinizing hormone release in camelids mediated at the hypothalamus? Reproduction. (2023) 165:395–405. doi: 10.1530/REP-22-0331, PMID: 36757313

[46] Ratto MH Paiva L Carrasco R Silva ME Ulloa-Leal C Ratto VF . Review: Unveiling the effect of beta-nerve growth factor on the reproductive function in llamas and cows. Animal. (2023) 17 Suppl 1:100754. doi: 10.1016/j.animal.2023.100754, PMID: 37567661

[47] Manni L Lundeberg T Holmäng A Aloe L Stener-Victorin E . Effect of electro-acupuncture on ovarian expression of alpha (1)- and beta (2)-adrenoceptors, and p75 neurotrophin receptors in rats with steroid-induced polycystic ovaries. Reprod Biol Endocrinol. (2005) 3:21. doi: 10.1186/1477-7827-3-21, PMID: 15941472 PMC1175857

[48] Fu H Sun J Tan Y Zhou H Xu W Zhou J . Effects of acupuncture on the levels of serum estradiol and pituitary estrogen receptor beta in a rat model of induced super ovulation. Life Sci. (2018) 197:109–13. doi: 10.1016/j.lfs.2018.02.005, PMID: 29421437

[49] Johansson J Redman L Veldhuis PP Sazonova A Labrie F Holm G . Acupuncture for ovulation induction in polycystic ovary syndrome: a randomized controlled trial. Am J Physiol Endocrinol Metab. (2013) 304:E934–43. doi: 10.1152/ajpendo.00039.2013, PMID: 23482444 PMC4116535

[50] Magarelli PC Cridennda DK Cohen M . Changes in serum cortisol and prolactin associated with acupuncture during controlled ovarian hyperstimulation in women undergoing *in vitro* fertilization-embryo transfer treatment. Fertil Steril. (2009) 92:1870–9. doi: 10.1016/j.fertnstert.2008.10.067, PMID: 19118825

[51] Angelopoulos N Goula A Tolis G . The role of luteinizing hormone activity in controlled ovarian stimulation. J Endocrinol Invest. (2005) 28:79–88. doi: 10.1007/BF03345534, PMID: 15816376

[52] Mercé LT Garcés D Barco MJ de la Fuente F . Intraovarian Doppler velocimetry in ovulatory, dysovulatory and anovulatory cycles. Ultrasound Obstet Gynecol. (1992) 2:197–202. doi: 10.1046/j.1469-0705.1992.02030197.x, PMID: 12796972

[53] Liu X Shi W Liu Z Shi S Ke C Zhang P . Effects of acupuncture on Luteinized Unruptured Follicle Syndrome: A meta-analysis of randomized controlled trials. Complement Ther Med. (2020) 49:102319. doi: 10.1016/j.ctim.2020.102319, PMID: 32147029

[54] Pastore LM Williams CD Jenkins J Patrie JT . True and sham acupuncture produced similar frequency of ovulation and improved LH to FSH ratios in women with polycystic ovary syndrome. J Clin Endocrinol Metab. (2011) 96:3143–50. doi: 10.1210/jc.2011-1126, PMID: 21816787 PMC3200239

[55] Stener-Victorin E Waldenström U Tägnfors U Lundeberg T Lindstedt G Janson PO . Effects of electro-acupuncture on anovulation in women with polycystic ovary syndrome. Acta Obstet Gynecol Scand. (2000) 79:180–8. 10716298

[56] Sox HC Lewis RJ . Pragmatic trials: practical answers to “Real world” Questions. JAMA. (2016) 316:1205–6. doi: 10.1001/jama.2016.11409, PMID: 27654606

[57] Ford I Norrie J . Pragmatic trials. N Engl J Med. (2016) 375:454–63. doi: 10.1056/NEJMra1510059, PMID: 27518663

[58] Hopewell S Chan AW Collins GS Hróbjartsson A Moher D Schulz KF . CONSORT 2025 statement: updated guideline for reporting randomised trials. BMJ. (2025) 389:e081123. doi: 10.1136/bmj-2024-081123, PMID: 40228833 PMC11995449

