# Supplementary material for: Clinical evidence of acupuncture for luteinized unruptured follicle syndrome: a systematic review and meta-analysis of randomized controlled trials
Source: Front Endocrinol (Lausanne). 2025 Aug 29;16:1640820. doi: 10.3389/fendo.2025.1640820 (PMC12425743; doi:10.3389/fendo.2025.1640820)
Supplement: Supplementary file 1 [file Table1.docx]

**Search strategy**

① Wanfang database: 题名或关键词: (针刺 或 针灸 或 电针) 与 (未破裂卵泡黄素化综合征 或 卵泡不破裂综合征 或 LUFS)

② VIP database: K=(针刺 + 针灸 + 电针) * (未破裂卵泡黄素化综合征 + 卵泡不破裂综合征 + LUFS)

③ CNKI database: SU='针刺' + '针灸' + '电针' AND SU='未破裂卵泡黄素化综合征' + '卵泡不破裂综合征' + 'LUFS'

④ Scopus: TITLE-ABS-KEY( ( acupuncture OR electroacupuncture OR auricular acupuncture ) AND ( "luteinized unruptured follicle syndrome" OR LUFS OR "unruptured follicle syndrome" OR "luteinized unruptured follicle" ) ) AND ( LIMIT-TO ( DOCTYPE , "ar" ) OR LIMIT-TO ( DOCTYPE , "cp" ) )

⑤ PubMed: ( (acupuncture[MeSH] OR acupuncture[tiab] OR electroacupuncture[tiab]) ) AND ("luteinized unruptured follicle syndrome"[tiab] OR LUFS[tiab])

⑥ Cochrane Library: #1 (acupuncture OR electroacupuncture):ti,ab,kw

#2 (luteinized unruptured follicle syndrome OR LUFS OR "unruptured follicle syndrome")

#3 #1 AND #2 in Trials

⑦ Web of Science: TS=( (acupuncture OR electroacupuncture) AND ("luteinized unruptured follicle syndrome" OR LUFS OR "unruptured follicle") )

Refined by: DOCUMENT TYPES: (ARTICLE OR PROCEEDINGS PAPER)

⑧ Sinomed: 主题: (("针刺" OR "针灸" OR "电针" OR "耳针") AND ("未破裂卵泡黄素化综合征" OR "卵泡不破裂综合征" OR "LUFS"))

限定: 人类、中文、核心期刊

⑨ ClinicalTrials.gov: (acupuncture OR electroacupuncture OR auricular acupuncture OR "needle therapy")

AND ("luteinized unruptured follicle syndrome" OR LUFS OR "unruptured follicle syndrome" OR "follicle persistence" OR "ovulatory disorder*")
