# Supplementary material for: Clinical evidence of acupuncture for luteinized unruptured follicle syndrome: a systematic review and meta-analysis of randomized controlled trials
Source: Front Endocrinol (Lausanne). 2025 Aug 29;16:1640820. doi: 10.3389/fendo.2025.1640820 (PMC12425743; doi:10.3389/fendo.2025.1640820)
Supplement: Supplementary file 2 [file Table2.docx]

**Study characteristics**

| **Study** | **Year** | **Sample size (n)** | | **Treatment regimen** | |
| --- | --- | --- | --- | --- | --- |
|  |  | **Trial** | **Control** | **Trial** | **Control** |
| Feng (21) | 2025 | 46 | 46 | MA + single-dose intramuscular injection of 10,000 U HCG (Re-dose: 5,000 U HCC if no ovulation in 48 h) + 50 mg CC/day * 5 days (In cases of ovarian non-response, the dosage was incrementally escalated by 50 mg/day during subsequent treatment cycles, with a maximum daily dose of 150 mg) | single-dose intramuscular injection of 10,000 U HCG (Re-dose: 5,000 U HCC if no ovulation in 48 h) + 50 mg CC/day * 5 days (In cases of ovarian non-response, the dosage was incrementally escalated by 50 mg/day during subsequent treatment cycles, with a maximum daily dose of 150 mg) |
| Li (28) | 2022 | 36 | 36 | MA + single-dose intramuscular injection of 5,000 to 10,000 U HCG | single-dose intramuscular injection of 5,000 to 10,000 U HCG |
| Zhang A (29) | 2021 | 30 | 30 | MA + single-dose intramuscular injection of 10,000 U HCG when the dominant follicle exceeds 18 mm in diameter | single-dose intramuscular injection of 10,000 U HCG when the dominant follicle exceeds 18 mm in diameter |
| Zhang B (30) | 2021 | 20 | 20 | MA + CHM | CHM |
| Tang (31) | 2019 | 30 | 30 | MA + single-dose intramuscular injection of 10,000 U HCG when the dominant follicle exceeds 18 mm in diameter | single-dose intramuscular injection of 10,000 U HCG when the dominant follicle exceeds 18 mm in diameter |
| Xu (32) | 2018 | 32 | 33 | MA + single-dose intramuscular injection of 10,000 U HCG (Re-dose: 5,000 U HCC if no ovulation in 48 h) + CHM + 50 mg LE/day * 5 days | single-dose intramuscular injection of 10,000 U HCG (Re-dose: 5,000 U HCC if no ovulation in 48 h) + CHM + 50 mg LE/day * 5 days |
| Zhu (33) | 2018 | 44 | 44 | MA + single-dose intramuscular injection of 10,000 U HCG when the dominant follicle exceeds 18 mm in diameter + CHM | single-dose intramuscular injection of 10,000 U HCG when the dominant follicle exceeds 18 mm in diameter + CHM |
| Tang (23) | 2017 | 30 | 30 | MA + single-dose intramuscular injection of 10,000 U HCG when the dominant follicle exceeds 18 mm in diameter + CHM | single-dose intramuscular injection of 10,000 U HCG when the dominant follicle exceeds 18 mm in diameter + CHM |
| Zeng (20) | 2017 | 32 | 76 | EA | single-dose intramuscular injection of 5,000 to 10,000 U HCG |
| Xu (34) | 2017 | 39 | 37 | MA + single-dose intramuscular injection of 10,000 U HCG (Re-dose: 5,000 U HCC if no ovulation in 48 h) + CHM | single-dose intramuscular injection of 10,000 U HCG (Re-dose: 5,000 U HCC if no ovulation in 48 h) + CHM |
| Guo (35) | 2017 | 30 | 30 | MA | single-dose intramuscular injection of 10,000 U HCG when the dominant follicle exceeds 18 mm in diameter |
| Wang (36) | 2016 | 45 | 42 | MA + single-dose intramuscular injection of 10,000 U HCG (Re-dose: 5,000 U HCC if no ovulation in 48 h) + CHM | single-dose intramuscular injection of 10,000 U HCG (Re-dose: 5,000 U HCC if no ovulation in 48 h) + CHM |
| Sun (22) | 2015 | 31 | 30 | MA + single-dose intramuscular injection of 10,000 U HCG when the dominant follicle exceeds 18 mm in diameter + CHM | single-dose intramuscular injection of 10,000 U HCG when the dominant follicle exceeds 18 mm in diameter + CHM |
| Liu (37) | 2011 | 23 | 21 | MA | single-dose intramuscular injection of 10,000 U HCG when the dominant follicle exceeds 18 mm in diameter |
| Jin (38) | 2005 | 35 | 30 | EA + single-dose intramuscular injection of 5,000 U HCG when the dominant follicle exceeds 18 mm in diameter + 50 mg CC/day * 3 days | single-dose intramuscular injection of 5,000 U HCG when the dominant follicle exceeds 18 mm in diameter + 50 mg CC/day * 5 days |

**Abbreviations**: n, number; MA, manual acupuncture; EA, electroacupuncture; CHM, Chinese herbal medicine; HCG, human choriogonadotropin; LE, letrozole; CC, clomifene citrate;
