# Supplementary figures and images for: Clinical evidence of acupuncture for luteinized unruptured follicle syndrome: a systematic review and meta-analysis of randomized controlled trials
Source: Front Endocrinol (Lausanne). 2025 Aug 29;16:1640820. doi: 10.3389/fendo.2025.1640820 (PMC12425743; doi:10.3389/fendo.2025.1640820)

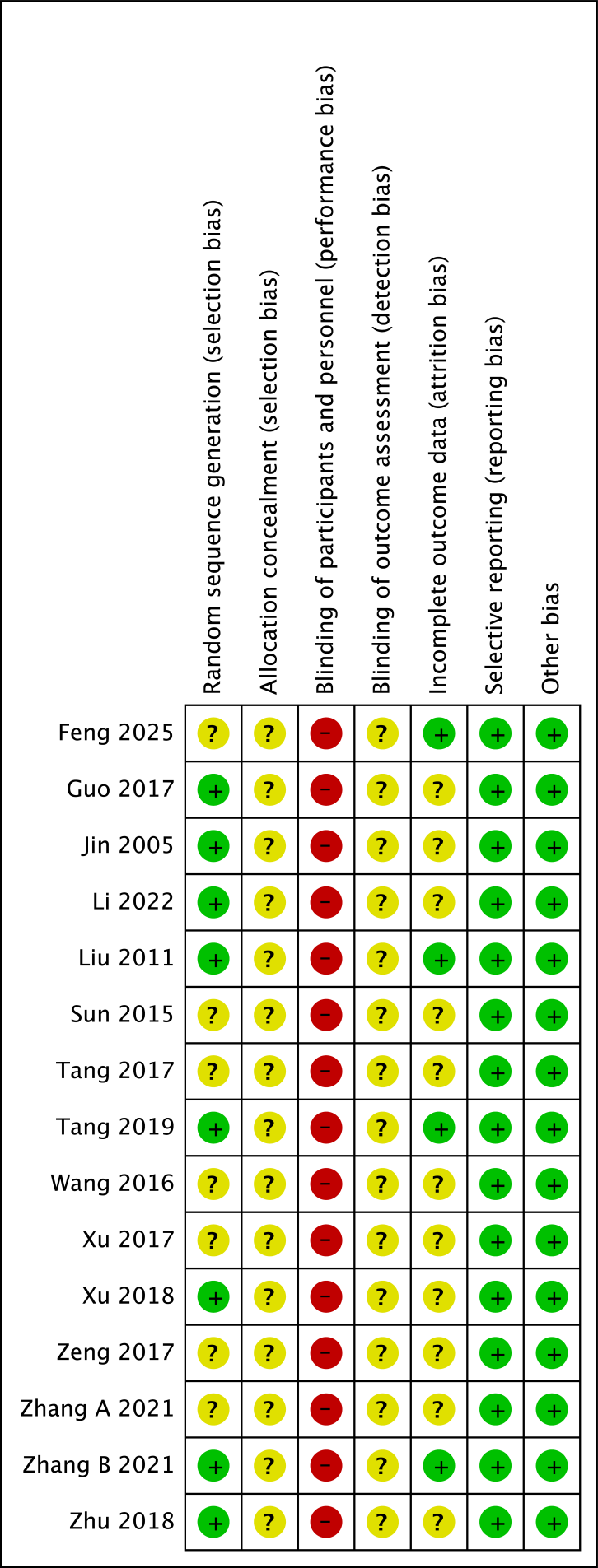


The risk of bias for each included study.

Supplement: Supplementary file 3 [file Table3.docx]
